# Umbrella-like systolic billowing of redundant mitral leaflets on left ventriculography in barlow mitral valve disease

**DOI:** 10.1093/ehjimp/qyag046

**Published:** 2026-03-11

**Authors:** Takeshi Nishi, Jagadeesh K Kalavakunta, Ashok Akula, Frank Saltiel

**Affiliations:** Department of Cardiology, Beacon Kalamazoo Hospital, 1521 Gull Road, Kalamazoo, MI 49048, USA; Department of Cardiology, Michigan State University, Lansing, MI, USA; Department of Cardiology, Beacon Kalamazoo Hospital, 1521 Gull Road, Kalamazoo, MI 49048, USA; Department of Cardiology, Michigan State University, Lansing, MI, USA; Homer Stryker MD School of Medicine, Western Michigan University, Kalamazoo, MI, USA; Department of Cardiology, Beacon Kalamazoo Hospital, 1521 Gull Road, Kalamazoo, MI 49048, USA; Department of Cardiology, Beacon Kalamazoo Hospital, 1521 Gull Road, Kalamazoo, MI 49048, USA; Department of Cardiology, Michigan State University, Lansing, MI, USA; Homer Stryker MD School of Medicine, Western Michigan University, Kalamazoo, MI, USA

An 81-year-old woman with a history of coronary artery disease, atrial fibrillation, and long-standing mitral valve prolapse presented with progressive exertional dyspnoea and palpitations. Cardiac examination revealed a late systolic murmur, prompting comprehensive imaging to assess mitral regurgitation (MR).

Transoesophageal echocardiography demonstrated bileaflet myxomatous prolapse with marked leaflet redundancy and thickening exceeding 3 mm. The mitral annulus was dilated with an average diameter >40 mm. Colour Doppler revealed three holosystolic MR jets (*Panel A* and Supplementary data online, *Video S1*), and quantitative assessment of the dominant jet showed an effective regurgitant orifice area of 0.45 cm^2^, consistent with severe MR. Multiplane imaging confirmed absence of mitral annular disjunction. Three-dimensional transoesophageal echocardiography further demonstrated complex multisegment systolic billowing, supporting a Barlow-type degenerative phenotype (*Panel B* and Supplementary data online, *Video S2*).

Left ventriculography, performed in the right anterior oblique 30° projection during invasive coronary assessment, provided an uncommon but striking depiction of global leaflet motion (see Supplementary data online, *Video S3*). In systole, markedly redundant mitral leaflets ballooned into the left atrium, forming a dome-shaped, umbrella-like multilobulated configuration (*Panel C*). In early diastole, the leaflet tissue returned toward the ventricle with restoration of coaptation (*Panel D*). No aortic regurgitation was identified on echocardiography. Although left ventriculography is no longer routinely used for evaluation of MR, this case illustrates its ability to visualize the integrated motion of excessive leaflet tissue in a manner complementary to echocardiography.

This image highlights a rarely documented ventriculographic appearance of advanced Barlow mitral valve disease and provides an intuitive visualization of leaflet excess that underlies severe degenerative MR.

**Figure:**
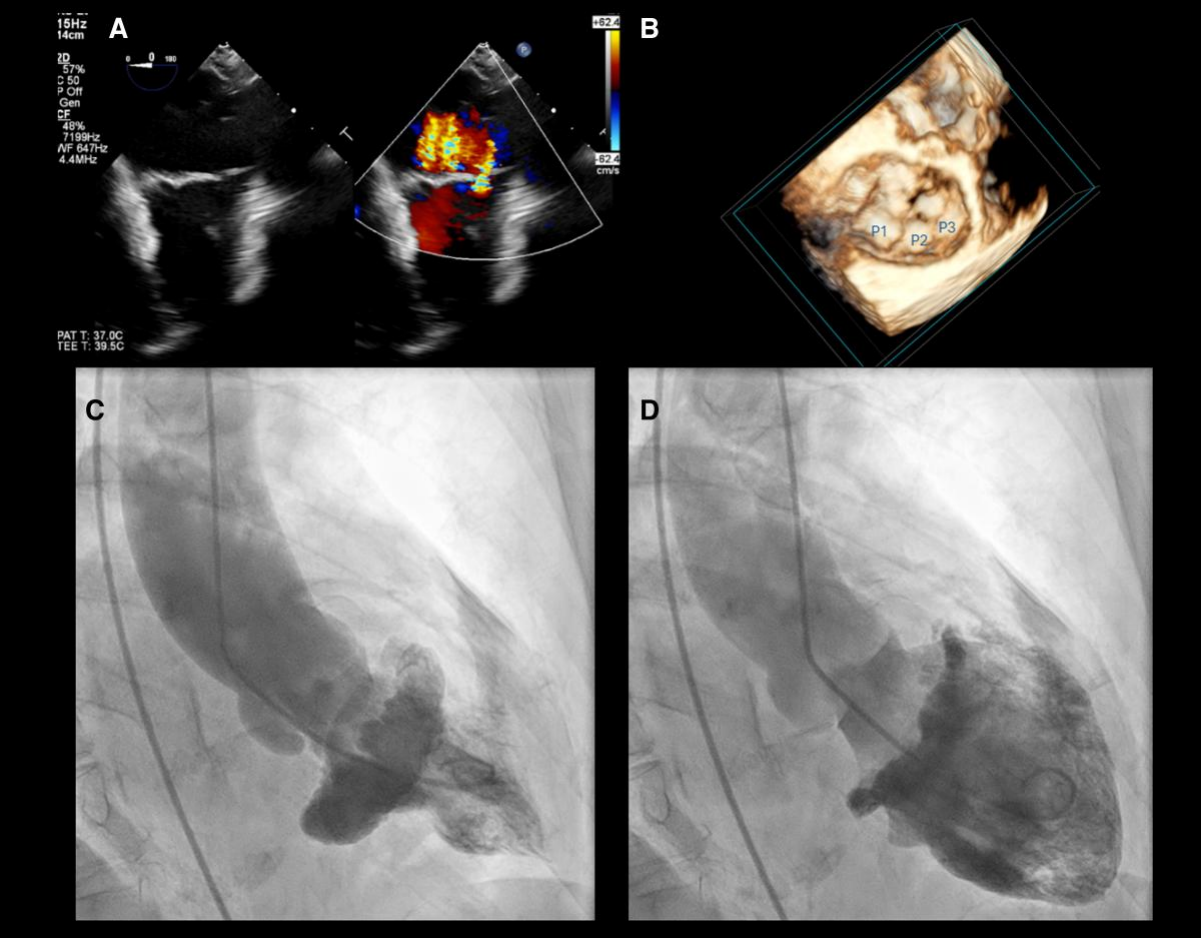


## Supplementary Material

qyag046_Supplementary_Data

## Data Availability

No new data were generated or analysed in support of this research.

## Lead author biography

Takeshi Nishi, MD, is an interventional cardiologist currently affiliated with Beacon Kalamazoo Hospital in Michigan, USA. He completed his cardiology training in Japan and received interventional cardiology training in both Japan and the United States. Dr. Nishi’s clinical and research interests focus on intracoronary physiology and imaging, structural heart disease, and advanced coronary interventions, with particular emphasis on the integration of multimodality imaging into procedural decision-making. He has authored multiple peer-reviewed publications in cardiovascular imaging and interventional cardiology.

