# Correction to: Umbrella-like systolic billowing of redundant mitral leaflets on left ventriculography in barlow mitral valve disease

**DOI:** 10.1093/ehjimp/qyag134

**Published:** 2026-08-10

This is a correction to: Takeshi Nishi, Jagadeesh K Kalavakunta, Ashok Akula, Frank Saltiel, Umbrella-like systolic billowing of redundant mitral leaflets on left ventriculography in barlow mitral valve disease, *European Heart Journal - Imaging Methods and Practice*, Volume 4, Issue 1, January 2026, qyag046, https://doi.org/10.1093/ehjimp/qyag046

The following change has been made to the originally published paper.

The first co-author was missing the assignment of an affiliation and this is corrected, so that the assignments in the author list now read: “Takeshi Nishi^1, 2^,” instead of: “Takeshi Nishi^1,^”.

